# Coronavirus Lockdown as a Major Life Stressor: Does It Affect TMD Symptoms?

**DOI:** 10.3390/ijerph17238907

**Published:** 2020-11-30

**Authors:** Sabina Saccomanno, Mauro Bernabei, Fabio Scoppa, Alessio Pirino, Rodolfo Mastrapasqua, Marina Angela Visco

**Affiliations:** 1 Orthodontic Residency, Department of Life, Health and Environmental Sciences, University of L’Aquila, 67100 L’Aquila, Italy; sabinasaccomanno@hotmail.it; 2 Department of Dental Clinic, Catholic University of Sacred Hearth, 00198 Rome, Italy; maurobernabei@outlook.it; 3 Faculty of Medicine and Dental Surgery, University of Rome “Sapienza”, 00185 Rome, Italy; fabioscoppa@chinesis.org; 4 Chinesis I.F.O.P. Istituto di Formazione in Osteopatia e in Posturologia, 00152 Rome, Italy; 5 Department of Biomedical Sciences, University of Sassari, 07100 Sassari, Italy; axelpir@uniss.it; 6 ENT Department, Rivoli Hospital, ASL TO3 Torino, Italy; rodolfomastrapasqua@gmail.com; 7 Faculty of Psychology, eCampus University, 22090 Novedrate, Italy

**Keywords:** COVID-19, lockdown, temporomandibular disorders, stress, orofacial pain

## Abstract

Temporomandibular disorders are multi-factorial conditions that are caused by both physical and psychological factors. It has been well established that stress triggers or worsens TMDs. This paper looks to present early research, still unfolding, on the relationship between COVID-19 as a major life stressor and TMDs. The main aims of this study were to: investigate the presence of symptoms related to TMDs and the time of onset and the worsening of painful symptoms in relation to the changes in social life imposed by the coronavirus pandemic; and to evaluate the perception of COVID-19 as a major stressful event in subjects who report worsening of painful TMD symptoms. One hundred and eighty-two subjects answered questionnaires—Axis II of the RDC/TMD, the PSS, and specific items about coronavirus as a stressful event—during the lockdown period for COVID-19 in Italy to evaluate the presence of reported symptoms of TMD and the level of depression, somatization, and stress perceived. The results showed that 40.7% of subjects complained about TMD symptoms in the past month. Regarding the time of onset, 60.8% of them reported that facial pain started in the last three months, while 51.4% of these subjects reported that their symptoms worsened in the last month and were related to the aggravation of pain due to the coronavirus lockdown as a major life event and to the stress experienced. The results of this study seem to support the hypothesis that stress during the pandemic lockdown influenced the onset of temporomandibular joint disorders and facial pain, albeit with individual responses.

## 1. Introduction

Temporomandibular disorders (TMDs) are multi-factorial conditions that are caused by both physical and psychological factors [1,2,3]. Several studies have examined the relationship between psychological stress and TMDs in the onset and chronicity of the musculoskeletal disorder [4,5,6,7].

Anxiety and stress increase the frequency, intensity, and duration of parafunctional habits, and are responsible for the hyperactivity of masticatory muscles and TMJ overload, facilitating the onset of the disorder [8]. Emotional stress has been shown to increase masticatory muscle activity, leading to the clenching of teeth and consequent circulatory changes in masticatory muscles, which can cause TMD symptoms [9]. De Paiva Tosato et al. [10] found that an increase in cortisol, which is a hormone released in stressful situations, was concomitant with greater muscle activity and TMD severity, with a positive correlation between electromyographic activity, salivary cortisol, the degree of temporomandibular disorder severity, and muscle activity.

Research has also suggested a relationship between negative effects and interference of TMD symptoms on daily life [11]. Because suffering associated with chronic pain is, in part, related to the meaning of pain to the individual, the actual intensity of the pain may increase in a stressful situation [12]. Lajnert et al. [9] found that female patients suffering from TMDs exhibit higher levels of depression, somatization, and anxiety compared to healthy peers, which indicates that physiological factors may play a predisposing role, in combination with a reduced level of body tolerance to pain and a decreased tolerance to stress. In patients with chronic painful TMDs, the intensity, duration, and localization of pain depend on the concurrent anxiety, depression, and somatization [13] that contribute to pain fixation [14].

In particular, several studies have pointed out the relationships between major life stressors and the occurrence and progression of temporomandibular disorders [3,15,16]. These studies show that patients with TMDs report more life stressors than healthy controls, even if a clear estimate of the total impact of such events has not been well-established in patients with orofacial pain [5]. It is considered that people exposed to stress are under increased risk of the occurrence and progression of TMDs, as stress and unpleasant life experiences are more frequent in patients with dysfunction [17,18,19,20]. Patients with a temporomandibular disorder report that their symptoms increase during stressful situations [21].

Some authors related TMDs with post-traumatic stress disorder (PTSD) syndrome, a form of pathological response to stress, during which individuals are permanently in a state of increased tension, continually re-living life-threatening experiences in their thoughts and dreams, to determine the frequency of subjective symptoms and clinical signs of dysfunction in the stomatognathic system among subjects suffering from PTSD. Some studies show that PTSD frequently occurs in patients with TMDs, and at the same time a higher TMD prevalence is found in PTSD subjects [22]. Uhac [20] found a correlation between war stress and temporomandibular disorder, with 82% of subjects in the group with PTSD having symptoms of dysfunction compared with 24% of the subjects in the healthy control group.

Patients with TMDs reported traumatic stressors of different natures: knowing that a family member or friend was injured or killed, experiencing automobile accidents, and suffering from violent attacks were the most commonly reported traumatic experiences and of personal significance for patients with TMDs [22,23,24,25]. Other significative life events defined as stressors and studied in relation to TMDs are typical life-changing events that can be perceived as stressors according to the various degrees of significance for the average individual [11,26,27].

Speculand et al. [18] found that patients with TMDs experienced twice as many undesirable stressful life events in the six months before onset as the control patients, and that life events contributed to the onset of TMD in almost 50% of the cases. Undesirable experiences are problems related to work, money, health, and other problems (e.g., bereavements, marital difficulties, serious arguments, or disappointments in life). These events were summarized by the author as “problems of loss and of interpersonal relationships”.

The recent period of lockdown imposed by COVID-19 in all of the European countries can be considered as a major life event that has affected millions. In Italy, the quarantine started on 22 February 2020 in some municipalities in North Italy. On 9 March, the government imposed lockdown measures on the whole country, which ended on 3 May. During this period, people were ordered to stay at home and socially isolate themselves to reduce the spread of the virus.

A preliminary recent study shows that the coronavirus outbreak is associated with a moderate to severe psychological impact in most individuals [28]. Female gender, student status, specific physical symptoms (e.g., myalgia, dizziness, colds, and respiratory inflammation), and poor self-rated health status were significantly associated with a greater psychological impact of the outbreak and higher levels of stress, anxiety, and depression.

To explore the relationship between TMDs and major stressful life events, we considered the recent lockdown due to the COVID-19 pandemic as a life stressor that may affect the onset or the worsening of orofacial pain related to TMDs. During the lockdown, people had to isolate themselves, and suddenly experienced a change to their personal, social, and working life that can be considered as a major life event. We assumed that the coronavirus outbreak may have caused “problems of loss and of interpersonal relationships” in people, as Speculand et al. [18] defined the stressful events they found related to the onset and worsening of TMD symptoms.

The coronavirus lockdown can be considered as a social stressor that impacted the lives of all of the people in quarantine in a similar way, so it has the unique characteristic to be a major event for everybody, and its different psychological impact can be evaluated in single individuals.

The aims of this study were: (i) to investigate the presence of symptoms related to TMDs, the time of onset, and the worsening of painful symptoms in relation to the change in social life imposed by the coronavirus pandemic. The hypothesis is that the lockdown is a major stressful event that may trigger painful temporomandibular disorders or increase their reported symptoms in subjects already suffering from this chronic dysfunctional pathology; (ii) to evaluate the perception of COVID-19 as a major stressful event in subjects who report a worsening of their painful TMD symptoms; and (iii) to compare levels of depression, somatization, and stress in subjects with reported TMD symptoms vs. the control group to examine if there is a significant difference. We assumed that the stressful event of a lockdown, impairing work and social life, may affect everybody’s level of depression, somatization, and stress, with an increase of these scales in the entire sample.

## 2. Materials and Methods

We adopted a self-administered online survey that includes the Research Diagnostic Criteria for Temporomandibular Disorders (RDC/TMD) Axis II, the Perceived Stress Scale (PSS), and specific items about coronavirus as a stressful event, to evaluate the presence of reported symptoms of TMDs and the levels of depression, somatization, and stress perceived in the previous month.

A snowball sampling strategy was utilized to recruit the subjects. The online survey was first disseminated to dental patients by emails and text messages, and they were encouraged to pass it on to others, including a non-clinical population. The survey was administered during the last part of the COVID-19 lockdown in Italy (18 April–3 May 2020) in order to evaluate the effect after the longest period of quarantine.

All participants provided informed consent and accepted the privacy policy for the protection of personal data before completing the survey. The questionnaire was administered by an online form service (Google Form service, Google LLC., 1600 Amphitheater Parkway, Mountain View, CA, USA).

The online survey was structured in the following four parts: (1) a first section for sociodemographic data; (2) a second section that consisted of Axis II of the RDC/TMD); (3) a third section in which the PSS scale was included; and (4) a fourth section in which additional items were asked.

### 2.1. Sociodemographic Items

Sociodemographic data were collected based on the gender, age, education, residential location, marital status, and employment status of the respondents.

### 2.2. Axis II of the RDC/TMD

Axis II of the RDC/TMD consists of a self-administered questionnaire that the subject completes, which contains specific items to assess the level of the patient’s chronic jaw pain, disability caused by their jaw complaint, depression, and nonspecific somatic symptoms [29]. Axis II of the RDC/TMD provides a reliable, valid assessment of psychosocial factors of TMDs including: pain intensity, pain-related disability, depression, and nonspecific physical symptoms [30]. It has proven to be a useful instrument for identifying TMD patients with high levels of distress, pain, and disability that can interfere with treatment response [31].

This instrument enables the severity of chronic pain to be rated by means of the Graded Chronic Pain Scale (GCPS), originally developed by Von Korff et al. [32,33]. The GCPS is composed of six items assessed on a 10-point scale, and one item on the number of disability days due to facial pain. The score categorizes pain in patients into five levels of chronic pain grades (0, no disability; 1, low disability, low pain intensity; 2, low disability, high pain intensity; 3, high disability, moderately limiting; and 4, high disability, severely limiting).

As for depression and somatization levels, the RDC/TMD Axis II enables their assessment by means of the depression and somatization scales of the Symptom Checklist 90-R (SCL-90-R), an instrument originally developed by Derogatis [34]. A total of 31 items were included in Axis II, belonging either to the Depression and Vegetative Symptom Scale (DEP) or to the Somatization Scale (SOM), which is here used to evaluate the presence of nonspecific physical symptoms, plus seven additional items added to the Depression and Vegetative Symptom Scale. The score rates patients as having normal, moderate, or severe levels of impairment in the depression and nonspecific physical symptom scales.

According to the literature [30], scores below 0.535 were considered normal, between 0.535 and 1.105 indicated moderate depression, and above 1.105 indicated the presence of severe ongoing depressive disorder. On the SOM scale, including the pain items, scores lower than 0.5 were considered normal, values between 0.5 and 1 indicated moderate somatization, and above 1 indicated severe somatization.

Only the subjects who reported TMD symptoms completed all of the items of the first part of Axis II, which provided specific information about myofascial pain, TMJ noise, and their interference in daily activities. All of the subjects in the sample completed the items of the Depression and Vegetative Symptom Scale (DEP) and of Axis II of the Somatization Scale (SOM).

### 2.3. PSS (Perceived Stress Scale)

This 10-item scale assesses the perception of stress, that is, the degree to which an individual appraises situations as stressful. The questions in the PSS asked about feelings and thoughts during the last month. In each case, respondents are asked how often they felt a certain way. PSS scores are obtained using Likert scale (0 = never, 1 = almost never, 2 = sometimes, 3 = fairly often, and 4 = very often). Responses are reversed (e.g., 0 = 4, 1 = 3, 2 = 2, 3 = 1, and 4 = 0) to the four positively stated items (items 4, 5, 7, and 8) and then summed across all scale items. Internal consistency is good, with a Cronbach’s alpha of 0.84 or greater, and constructed validity has been demonstrated, as the PSS correlates significantly with other measures of stress appraisal.

### 2.4. Additional Items

Additional items included specially designed items to investigate the impact of the coronavirus lockdown and its perception as a major stressful event that can be related to the worsening of TMD symptoms.

These items are: (1) “In the past month has your facial pain worsened, improved or remained constant?” (2) “If worsened, do you think there have been stressful events/situations recently that have led to a worsening of your pain?” and “If yes, can you possibly specify which one(s)?”

### 2.5. Statistical Methods

Descriptive statistics were calculated for sociodemographic characteristics and additional health information variables.

Baseline demographic and pain duration features were compared among samples by using the chi-squared test and analysis of variance (ANOVA) with Bonferroni’s post hoc test, when needed. The relative and absolute frequencies of the different scores for the Graded Chronic Pain Scale (GCPS), the Depression and Vegetative Symptom Scale (DEP), and the Somatization Scale (SOM) in the study population were described, and these data were expressed as medians. Distribution of DEP and SOM scores between categories of patients identified by the GCPS items were assessed by means of Kruskal–Wallis. The null hypotheses were that no differences existed between GCPS categories in terms of DEP and SOM scores. To test for the influence of pain duration on the degree of pain-related disability and DEP/SOM levels, the chi-squared test was performed to compare the prevalence of the different GCPS, DEP, and SOM categories between patients with pain lasting for about six months. Again, the null hypothesis was that no differences emerged among patients with pain for more or less than six months regarding the prevalence of the different degrees of pain-related impairment, depression, and somatization. There was not a significant difference on any scale between those who experienced pain during the previous months and those who did not, as the number of people who responded to this question was too small.

Statistical significance was set at *p* < 0.05. All of the statistical procedures were performed with the Statistical Package for the Social Sciences (SPSS 25.0, SPSS Inc., Chicago, IL, USA).

## 3. Results

One hundred and eighty-two subjects (52 males and 130 females, median age group = 45 years) answered the online survey.

The majority of respondents were women (71.4%), married (76.4%), and had at least a bachelor’s degree (52.2%). The age of the subjects was quite evenly distributed between 30 and 60 years of age (Table 1). Most respondents to the survey resided in Central Italy (73.1%), followed by Southern Italy or islands (14.8%), and Northern Italy (12.1%). The majority of the respondents were employed (83%).

Forty point seven percent (40.7%) of subjects complained of pain in the face, jaw, or temples in the past month (74 of 182), and 74.3% of them were women. Among other reported symptoms, almost all of the 74 respondents (68 subjects) reported complaints related to bruxism (grinding of teeth or clenching of the jaw during the day and/or night, and jaw ache or feeling stiff when waking up in the morning), and 66 subjects reported migraines as well.

The most frequent physiological activities prevented or limited by the present jaw condition were: yawning (72.7%), chewing (51.5%), swallowing (42.4%), eating hard foods (40.9%), and talking (34.8%). Twenty-seven subjects (42.2%) reported four or more of these and/or other symptoms that limited their daily activities, and we considered them as subjects with severe TMD.

The GCPS scores allowed detection of high disability with severely limiting pain-related impairment (grade IV), in 37.8% of 74 subjects, and high disability with moderately limiting impairment (grade III) in 16.2% of subjects.

In the overall sample, the prevalence of severe depression and somatization was 53.3% (97 subjects) and 58.8% (107 subjects), respectively; 23.1% of respondents (42 subjects) showed a moderate depression score, and 20.9% of them (38 subjects) showed a moderate somatization score. In the group with TMD symptoms, 47.3% of respondents reported a severe level of depression (35 subjects), and 51.4% of them reported a severe somatization score (38 subjects).

Concerning the results of Perceived Stress Score (PSS), a total of 44 subjects reported low perceived stress, while 80 subjects reported a moderate level of stress and 58 a severe stress. Low stress perceived was reported by 24/74 (32%) of the subjects who reported TMJ pain vs. 20/108 (18%) who did not report TMJ pain; moderate stress was reported by 32/74 (43%) vs. 48/108 (44%), and severe stress by 18/74 (24%) vs. 40/108 (37%).

Data recorded showed a correlation between pain-related disability (GCPS categories) and both depression and somatization (DEP and SOM), and a strong correlation with perceived stress score (Table 2).

Regarding the comparison between groups, no association was found between depression and somatization scores and the presence of reported TMD symptoms. On the DEP scale, 23% of all of the respondents (42 subjects) had a moderate level of impairment in depression, and 53.3% were at the severe level. On the SOM scale, excluding the pain items, 20.9% (38 subjects) had a moderate level of somatization, and 58.8% (107 subjects) had severe somatization. In the group with TMD-reported symptoms, 47.3% of them had a severe level of depression, and 51.4% had severe somatization.

The PSS scores instead showed a strong correlation with the presence of reported TMD symptoms and distribution differences between the TMD and non-TMD group in terms of stress class, which showed statistical significance (*p* < 0.05) (Table 3).

Regarding the time of onset of TMD symptoms, 60.8% of the subjects reported that facial pain started for the first time in the last three months (about three month ago = 21.6%, or 16 subjects; about one month ago = 39.2%, or 29 subjects), with orofacial pain defined in the majority of cases as recurrent (67.6%, or 50 subjects). Many of them (62.2%, or 46 subjects) have never been to a dentist before for facial aches or pain, so we can suppose that their symptoms were never diagnosed as TMDs.

Of the 74 subjects who reported facial pain, 51.4% reported that their symptoms worsened in the last month. Ninety-four point seven percent (94.7%) of them (35 subjects) reported that aggravation of the pain was related to a recently occurring major life event. When asked to identify the stressful event that affected them in the last period and caused their pain symptoms, 16 subjects referred in a generic way to coronavirus. Seventeen of them (48.6%) related the worsening of pain more specifically to the limitations imposed by the lockdown, with the obligation to stay at home and the loss of their interpersonal relationships. Some of the expressions used to describe the stressful event were “segregation at home”, “stuck at home”, and “residence requirement”. Two of the respondents related the worsening of their pain to the “loss” of scheduled dental visits.

Twelve subjects reported a lower intensity of their symptoms in the last month, while 24 subjects reported that the pain intensity remained constant.

**Figure 1:**
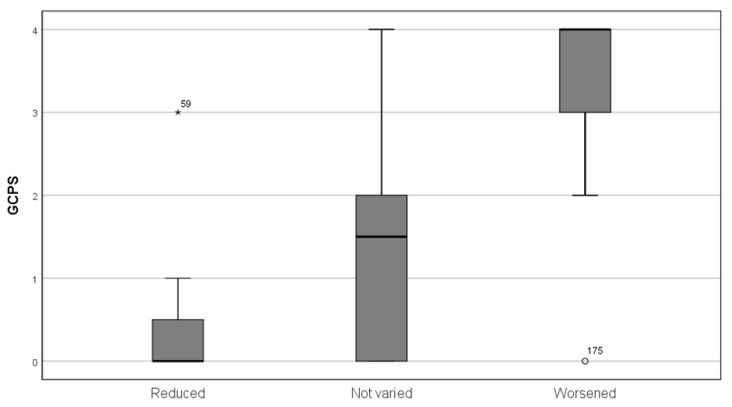
Graded Chronic Pain Scale (GCPS) class and pain variation in the last month. The main box represents the interquartile range. The black line represents the median and the confidence interval. Subjects #59 and #175 were outliers.

**Figure 2:**
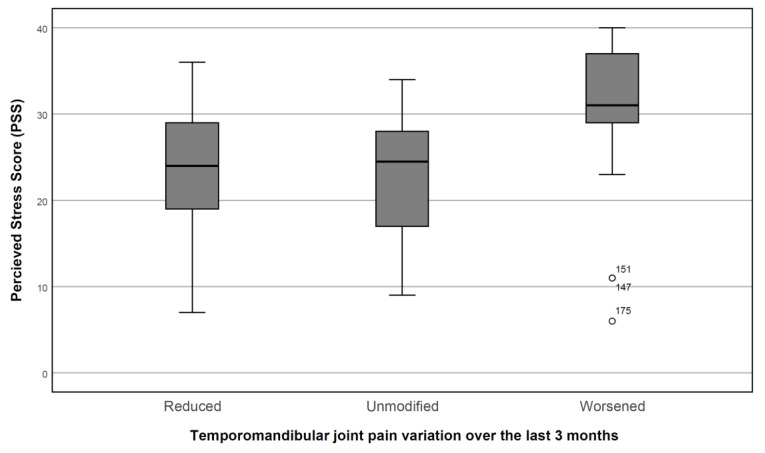
Perceived Stress Scale score (PSS) in relation to temporomandibular joint pain variation over the last three months. The box represents the interquartile range, and the black line represents the median and the confidence interval. Subjects #147, #151, and #175 showed outlier values.

The 38 subjects that reported a worsening of TMD symptoms during the quarantine showed a significantly higher depression score (2.53 + 0.85 vs. 1.87 + 1.63, *p* < 0.05), a non-significant mean somatization score (2.10 + 0.76 vs. 1.90 + 1.21, *p* > 0.05), and a significantly higher GPCS class (median 4 vs. 3, interquartile range = 1, *p* < 0.01) (Figure 1); the age group is not significant (median age group 40–60, range = 2, *p* > 0.05), but those whose pain had worsened showed lower education (median = high school for worsening patients, college education for the other groups, *p* < 0.01). Sex was not a significant predictor of worsening conditions. The score of PSS was found to increase in the subject that reported a worsening of their symptoms in the last three months (Figure 2).

Twelve subjects reported a lower intensity of their symptoms in the last month. These subjects showed low depression (mean = 2.04 + 1.79), somatization (mean = 1.79 + 1.33), and GPCS (median = 0, maximum = 3) scores, with no prevalence of sex or age affecting the entire study population (median age group = 3.50).

## 4. Discussion

The results highlight the impact of COVID-19 on the perception of pain affecting the craniofacial complex.

Of 74 subjects that reported TMD symptoms, as many as 60.8% of them reported a recent onset of facial pain: for 21.6% of them (16 subjects), symptoms started for the first time in the last three months, while they started about one month prior for 39.2% (29 subjects). Moreover, of the 74 subjects, 51.4% reported that these symptoms worsened in the last month. It appears significant that 94.7% of them (35 subjects) reported that the aggravation of the pain was due to the stressful lockdown and its consequences. Some of the expressions used by the respondents to describe the recent stressful experience were “segregation at home”, “stuck at home”, and “residence requirement”, which gives the idea of how traumatic the lockdown was for some of the respondents to the questionnaire.

These data seem to support the hypothesis that the lockdown was a major life event that may have triggered or increased orofacial pain and temporomandibular symptoms. This is in line with previous studies that reported the impact of more recent stressful life events in patients with myofascial pain than in control patients [5,35,36]. Some authors [36,37] related long-term stress (i.e., financial or job problems) more to joint or muscular pain, and short-term stress (i.e., self-health and problems of interpersonal relationships or loss) to myofascial pain, while other researchers did not observe psychological differences between patients with myofascial pain and patients with joint pain [38].

It is also interesting that two of the respondents related specifically the worsening of their pain to the “loss” of scheduled dental visits. This seems to suggest the importance of developing an alternative way to follow-up with patients at a distance, such as tele-medicine [39].

The subjects that reported a worsening of TMD symptoms during the quarantine showed a significantly higher depression score, a significantly higher PSS score, and a significantly higher GPCS class. Therefore, these subjects seem to be characterized by greater depressive symptoms, greater stress experienced, and greater painful symptoms affecting the temporomandibular joint.

However, by analyzing the results, we found a minority of patients (12 people) with an improvement in pain symptoms during the lockdown. These subjects showed low depression (mean 2.04 + 1.79), somatization (mean 1.79 + 1.33), and GPCS (median = 0, maximum = 3) scores. In the absence of any specific therapy, an explanation may be linked to the specific lifestyle that each participant in the study led before the lockdown.

It is relevant to highlight that the scores on the scales are high throughout the sample, to underline how much the stressful events linked to the lockdown had a significant impact on all subjects. This is in line with a recent study that showed relatively high rates of depression symptoms, anxiety symptoms, and perceived stress related to the COVID-19 outbreak and lockdown measures in the general population in Italy. These outcomes were associated with a number of COVID-19 related risk factors, including being under quarantine, working activity discontinued due to lockdown measures, or experiencing other stressful events (working, financial, or relationship problems) [40]. Therefore, we can consider the coronavirus lockdown as a major event that may include different types of stressful events (financial, job, relationship, health) at the same time, and the psychological impact of these events can be evaluated individually.

Unlike the stress scale, which has values that are significantly higher in subjects with TMD, the DEP and SOM scales do not seem to be able to significantly discriminate subjects with TMD compared to those who do not have this symptomatology.

In this direction, De Resende et al. [41] found that anxiety showed greater strength of association with TMD, with an OR greater than five times more for individuals with anxiety to have TMDs, when compared to those with normal levels of anxiety. A similar finding was reported by De Lucena et al. [42], who observed that 153 students who had gone through a stressful event were more likely to have TMDs (with OR = 2.6) in the three months after university admission examinations, while having an OR = 5.6 at baseline. Other research suggested that pain chronicity in TMDs may be related to an increase of psychological distress [43,44].

We find that both DEP and SOM scores have a significant relationship with GCPS ratings. The prevalence of severe depression increased with the rate of pain-related impairment, thus supporting the early view that the three main components of the RDC/TMD Axis II are related with each other [30,45].

Moreover, the result of the present study confirms that the female gender is an important factor in the etiology of TMDs [46], as 74.3% of subjects who complained of pain in the face, jaw, or temple in the past month were women. Several authors already argued that TMD is mostly seen in females because of hormonal, postural, emotional, and functional factors, in addition to the muscular structure and genetic predisposition [47,48,49,50]. Recent studies showed also that the prevalence of anxiety, depression, and stress during the COVID-19 pandemic was higher in woman than in men [51,52,53,54]. Therefore, we can hypothesize that women are more exposed to the development of pain symptoms of TMD in response to the stress experienced during the pandemic.

Finally, in our study, the effect of age on muscle pain disorders seemed to be non-significant [55].

This study has some important limitations due to the sampling technique that could have introduced important selection bias, as suggested by the highly unbalanced gender ratio and wide range of age observed.

Another important limitation is that the study relied on a fairly small group of subjects. It will be interesting in the future to see an aggregate of data coming from other studies that focus on the impact that the pandemic had on people with and without TMD and facial pain.

Moreover, clinical symptoms of TMDs were assessed only by a self-assessment scale. The data that emerged should naturally find a response from the clinical analysis to confirm the diagnosis of TMDs. Furthermore, the symptoms reported by the subjects may not necessarily remain in the following months, considering that the lockdown is configured as a transitory event. What appeared significant was the possibility of verifying the effect of a stressful event on the pain related to the temporomandibular joint, evaluating it at the very moment in which the event is experienced. In any case, what seems really significant is the perception that the subjects have of the TMD, which shows how the psychological component related to stress is implicated in the amount of pain perceived by the subjects.

## 5. Conclusions

Over the years, evidence has been growing that the psychosocial aspects in a TMD assessment are important for predicting a treatment outcome [56,57], thus lending support for the need for a thorough psychosocial assessment of TMD patients.

The results of this study seem to support the hypothesis of a lockdown as a major stressful event that may trigger temporomandibular disorders or increase their reported symptoms in subjects already suffering from this dysfunctional pathology. Almost all of the 51.4% of subjects who reported a worsening of TMD symptoms in the last month related this condition to the coronavirus lockdown and to the stress experienced in that period. These subjects seem to be characterized by greater depressive symptoms, greater stress experienced, and greater painful symptoms affecting the temporomandibular joint.

However, we can affirm that beyond the general concern linked to the infection of COVID-19, the lockdown was not a stressful event for everyone, depending on the individual’s psychological characteristics and lifestyle. This could explain how painful symptoms during the lockdown period actually improved in a small group of patients.

The DEP and SOM scales did not effectively distinguish the clinical group from the control group. The scores on these scales are high throughout the sample, to underline how much the stressful events linked to the lockdown had a significant impact on all subjects. Instead, the stress scores obtained with PSS distinguished the subjects of the two groups, and those with TMDs tended to report a greater perceived stress. This result suggests that the stress scale can be considered a useful assessment tool for TMDs to be combined with Axis II.

## Figures and Tables

**Table 1:** Demographic data of respondents. Age is expressed in years.

Age Group	Frequency
<20	1 (0.5%)
20–30	28 (15.4%)
30–40	46 (25.3%)
40–50	44 (24.2%)
50–60	46 (25.3%)
60–70	17 (9.3%)
**Sex M/F**	52/130
**Education**	
Middle High	8 (4.4%)
High School	70 (38.5%)
College	95 (52.2%)
PhD	8 (4.4%)

**Table 2:** Correlation between pain-related disability (GCPS class) and depression (DEP), somatization (SOM), and perceived stress score (PSS).

GPCS Class	Median Perceived Stress Score (PSS)	Median Depression Score (DEP)	Median Somatization Score (SOM)
0	21.0	1.4	1.5
1	25.0	1.4	2.0
2	19.5	0.8	1.2
3	30.0	2.5	2.3
4	33.0	2.8	2.33
Significance	*p* < 0.05	*p* < 0.05	*p* < 0.05

**Table 3:** Correlation between TMJ pain in the last six months and PSS, DEP, and SOM scores. Expressed as mean + SD.

TMJ Pain in the Last Six Months	Median Perceived Stress Score (PSS)	Median Depression Score (DEP)	Median Somatization Score (SOM)
No	23.0 ± 9.1	1.3 ± 1.2	1.2 ± 1.4
Yes	18.0 ± 9.6	1.1 ± 1.4	0.9 ± 1.0
Significance	*p* < 0.05	*p* < 0.05	*p* < 0.05
